# Lived experiences of students with virological failure on antiretrovirals at a university in Limpopo

**DOI:** 10.4102/curationis.v46i1.2478

**Published:** 2023-10-30

**Authors:** Mahlodi P. Maphakela, Mokoko P. Kekana, Eric Maimela

**Affiliations:** 1 Department of Public Health, Faculty of Health Sciences, University of Limpopo, Polokwane, South Africa

**Keywords:** virological failure, disclosure, stigma, antiretroviral drug packaging, supermarket approach

## Abstract

**Background:**

Human immunodeficiency virus (HIV)-positive students at a rural university in Limpopo province are followed-up according to the national guidelines for the treatment of HIV. Blood monitoring revealed that some students on antiretroviral (ARV) treatment were not virologically suppressed despite adherence and compliance being emphasised at every visit.

**Objectives:**

The study sought to identify the students’ experiences that were hindering the viral load from improving.

**Method:**

A two-phase qualitative, explorative, descriptive study design was followed. Convenience purposive sampling methods were taken on. By means of a semi-structured interview guide, face-to-face interviews were directed. Thematic content analysis was applied.

**Results:**

Non-disclosure, noisy ARV packaging, stigma, and service delivery played a role in determining levels of student adherence and compliance with ARVs in the study sample.

**Conclusion:**

Study findings suggest practical recommendations to improve compliance among students on ARVs: provision of HIV education to all students to help reduce stigma and make it easier to disclose HIV status; use of user-friendly noise-free packaging by pharmaceutical companies to enclose medication, such as blister packs; a supermarket approach in service delivery points to reduce the stigmatising effects of consulting rooms for ARV services.

**Contribution:**

There is scope to examine the relevance of these findings for other students in the country, to compare them, and to use material from larger studies to guide targeted interventions that could improve adherence among young people.

## Introduction

Highly active antiretroviral therapy (HAART) in the form of at least three antiretroviral (ARV) drugs (Efavirenz 600 mg, Emtricitabine 200 mg and Tenofovir 300 mg or Dolutegravir 50 mg, Lamivudine 300 mg and Tenofovir 300 mg) are only recommended to stop the advancement of human immunodeficiency virus (HIV) disease and maximally suppress the virus. The purpose of these drugs is to reduce the HIV viral load to undetectable limits (Trotta et al. 2010). These drugs are not a cure to the virus but radically reduce the amount of virus in the body to prevent the virus from destroying the immune system (WHO 2010). When taken reliably and correctly, these drugs can reestablish the immune system, prevent HIV transmission, decrease HIV-related illnesses and death, and improve their quality of life.

Human immunodeficiency virus-positive clients are dependent on their use of these ARV drugs every day, at the same time, for the rest of their lives, unless a cure is found. The drugs may cause resistance and increase the risk of failure of treatment if not taken as such (WHO 2010). A study conducted in Wales by Bravo et al. (2010), on hard decisions faced by people living with HIV, found that they experienced tough psychosocial difficulties in wide ranging areas including disclosure, stigma, social support, adherence, decisions about sexual activity and yearnings about parenthood, which prevent them from taking treatment as prescribed.

Globally there is a serious concern about new infections and deaths among youth and adolescents, and the number of people dying from AIDS-related diseases tripled between 2000 and 2015, the only age group to have experienced a rise in those years. In 2016, 55 000 adolescents died from AIDS-related causes worldwide. AIDS is now the leading cause of death among young people in Africa and the second leading cause of death among young people worldwide (Avert 2017).

The youths and adolescent groups are particularly vulnerable to HIV because of the physical, social, psychological, and economic attributes of adolescence, which include exploring and engaging in high-risk sexual behaviours. Peer pressure also plays a role; for example, many young women desire to acquire extravagant items, which may encourage them to practise unsafe sex practices (Asante & Oti-Boadi 2013).

The estimated number of young people infected with HIV in South Africa in 2017 was approximately 870 000, but only 10% of those infected were virally suppressed (Pillay et al. 2020). To try to understand the reasons, a small-scale qualitative study was conducted with university students on ARVs, who were found on routine monitoring to have virological and immunological failure. The study then aimed to explore their experiences as a starting point for designing strategies that could reduce or prevent treatment failure, resistance, and mortality.

### Problem statement

Human immunodeficiency virus positive students at a university in Limpopo province are tested, initiated on ARVs and all their follow-up treatments, blood routines, and care happen at the university’s health and wellness clinic. They are first monitored for their viral load 6 months after initiation of ARVs, and this is according to the Department of Health guidelines for follow-up and treatment. Routine blood monitoring and student ARV files revealed that in some patients, the viral load was not suppressing. This observation motivated the decision to explore possible reasons for these unexpected outcomes in this young student population. There is literature reported on the experiences of adults with ARVs and adherence (Musumari et al. 2013; Nagata et al. 2013), to name a few, but less is known about the experiences of youth and adolescents on ARVs, especially those attending higher education institutions. Therefore, the present study aimed to investigate and describe the experiences of a sample of students on ARVs, whose viral load was not suppressing as expected. Understanding and identifying what hindered virological success could help in designing better and more targeted strategies to prevent treatment-failure and resistance to ARVs among student populations.

### Objective

The study sought to identify those HIV-positive students’ experiences that were hindering the viral load from improving while on ARV drugs.

## Method

### Research design

A two-phase qualitative, exploratory, and descriptive research design was undertaken (Creswell 2012), enabling the researcher to explore in depth the lived experiences of the participants in two sets of conditions. The first phase was conducted in 2018, during the unrestricted social and living conditions before the coronavirus disease 2019 (COVID-19) pandemic gave rise to the restrictions of the National State of Disaster; the second follow-up phase was conducted in 2022 after the restrictions were lifted. This process allowed the study to examine whether or not the experiences of the participants differed in terms of changed circumstances because of the pandemic.

### Research instrument

A semi-structured interview guide was used in both phases to examine and report on the lived experiences of students on ARVs who were found to be having virological failure. The interview method yields understanding of values, beliefs, practices, and perceptions of a selected population group through asking questions and probing.

### Study context

The study took place at one rural university in Limpopo province, which is located 40 km, east of the provincial capital town of Polokwane. The university’s health and wellness clinic is on the track of the popular Gate 2 that students use when going to local shops, east of the university making it visible and accessible. It offers services to all registered students, which number approximately 21 000 as well as 890 staff members. The health and wellness clinic offers Primary Health Care (PHC) services including HAST (HIV and/or AIDS, sexually transmitted infections and tuberculosis) services, assisting these registered students to avoid having them attend to local community clinics, having to queue in long lines, and having them miss classes in turn. The clinic operates according to the university’s operational times: Monday to Friday from 07:30 to 16:00, which are the days and times when students will be attending class.

### Population and sampling

The target population consisted of undergraduate and postgraduate HIV-positive students who had been on ARVs for more than a year. A convenient purposive sampling was used for the study and only those students found on routine surveillance to have virological failure were interviewed.

#### Phase 1

The student sample in the first phase of the study, conducted before the onset of the COVID-19 pandemic in 2018, consisted solely of 10 students on ARVs who were found to have virological failure.

#### Phase 2

The follow-up study, after the easing of COVID-19 lockdown restrictions, focused on the students who had participated in the first pre-pandemic phase 3 years earlier and were still available for interviews. Only 3 out of the 10 students were interviewed, 4 were no longer in the system as they graduated and the other 3 were no longer available for the study, hence only 3 students were interviewed for phase 2.

### Data collection and procedures

#### Phase 1

In the study, data were collected using a semi-structured interview guide to explore and describe the experiences of the selected sample of students on ARVs who met the criteria. The interviews lasted 30–45 min. During the discussions with the students, the first author probed for more information and understanding, and encouraged the participants to talk freely. The interviews were recorded and field notes were taken with the permission of the participants.

#### Phase 2

The interview guide from Phase 1 was used to obtain follow-up data from participants to establish their experiences during the time of COVID-19 pandemic restrictions. The themes and sub-themes in phase 2 were reduced as other variables that were factors affecting them at the university were no longer a factor in their home environments, hence interviews only lasted for 10–20 min.

### Data analysis

The purpose of data analysis is to organise, provide structure, and elicit meaning from the data (Polit & Beck 2012). All the recordings were transcribed verbatim. The Tesch method was used to analyse and to interpret the data, and make sense of the information obtained (Creswell 2003).

This meant re-reading through the raw data in the material and make clarity of meaning filtering for substantial points. Themes were formed, clustered together and coded. The analyst then went through all the transcripts and marked the statements with the codes assigned to each of the themes. The themes were then compared with another independent analyst, and similarities and differences were discussed until consensus was reached on the final themes.

### Trustworthiness

To confirm the accuracy of the data obtained in this investigation, the Lincoln and Guba model for determining trustworthiness was applied. The model considers four elements: credibility, dependability, confirmability, and transferability (Polit & Beck 2012). Credibility was achieved by engaging with the participants, building rapport, and using an independent co-coder for data analysis. Reliability was achieved by comprehensively documenting each step of the research to provide future researchers with evidence for the study. The researcher verified confirmability by storing the field notes and audiotape recording in a secure location where they could not be accessed or altered. Transferability was achieved through data saturation, description of the methodology, and findings, and supporting verbatim quotations from the participants’ responses.

## Findings

The study was conducted at a rural university in Limpopo Province, South Africa. There were 10 participants interviewed in phase 1 (8 females and 2 males) and these were participants whose Viral load remained unsuppressed after taking ARVs for more than a year.

These participants were registered students at the rural university, from 1st year to Honours level. Their ages ranged from 21 years to 32 years. Three of the participants were married (2 females and 1 male). In phase 2, only 3 females from the original number took part and shared their experiences of the subject matter under the strict lockdown conditions. The data collected from these participants were analysed with the generated themes and categories. The generated themes and categories reflect the views of the participants and were presented with the concurrent support of direct quotations.

### Phase 1

Twelve participants who have been on ARVs for more than a year and had virological failure, according to records, were sampled; but only 10 consented to be interviewed. In this phase, the data collected represents social conditions that can be regarded as the ‘old normal’ ones before the period of COVID-19 restrictions. All 10 participants were interviewed as the available subjects and sharing their different experiences on the subject matter. Based on their responses, the researcher identified five themes. But only three key themes are presented because of the changed circumstances, pre and post COVID-19 restrictions since studying and side effects were no longer factors to be considered.

#### Theme 1: Disclosure versus stigma, and difficulties with treatment when among people

Because they worry about being stigmatised and excluded, participants said that disclosing their HIV positive status was difficult. According to one of the students:

‘… in class, there are discussions on information sharing, you find that people say a lot bad about HIV information statistics and so on. So, it is clear to my mind that it says, if people can understand or know about my status, maybe they will stigmatize me or they will run away from or be disassociated with me.’ (P1, 22 years, Female, 2nd level, Single)

They found it challenging to receive treatment when around peers because of their fear of disclosure. Three subthemes emerged from this theme: failure to disclose among roommates, failure to disclose among family members, and fear of partner rejection after disclosing HIV status.

**Sub-theme 1.1: Difficulties adhering to therapy because of lack of disclosure between roommates:** Participants who shared rooms with other students found it challenging to disclose their status to their roommates, leading them to try to conceal the fact that they were taking medication or skipping it when others were around:

‘No, my roommate and even friends don’t know about my status. Yes, I remember this other day when I visited my friend and it was half past eight, so I had to take my medication, I just pretended as if I was taking a tissue and I put that pill there, I pretended as if I wanted to spit out something …’ (P6, 28 years, Female, 4th level, Engaged)

‘When she [*my roommate*] is in the room, I don’t take my medication.’ (P9, 21 years, Female, 3rd level, Single)

**Sub-theme 1.2: Lack of disclosure among family members affecting the taking of treatment at home;** The HIV-positive students of this study found it challenging to receive therapy at home because of misunderstandings among family members. One of them said that while it was simpler to continue treatment after disclosing her status to her friends, it was more challenging to do so to her family members, who did not understand and required more information about HIV:

‘But when it comes to family it was a bit difficult because of my cousins or my siblings they used to say, like make jokes about people who are HIV positive they will be like, ha that person is dying, oh you saw that person, oh sphamola se mo tshwere [*HIV has got her*], things like that and then it was difficult to tell them that do you see that person you are talking about, I am also like him or her.’ (P2, 32 years, Male, Honour’s degree, Single)

A couple of the participants specifically feared revealing their status to their mothers:

‘Yooh, at first I took time to reveal more, especially to my mother, I remember that I was pregnant at that time. And I couldn’t tell her *gore* [that] now I am pregnant and positive, so I didn’t disclose to her.’ (P4, 26 years, Female, 3rd level, Married)

‘Uhm in my family, nna my mother, eish my mother o, she is, *oa re roga* [insults us], *thata* [a lot].’ (P9, 21 years, Female, 3rd level, Single)

**Sub-theme 1.3: Fear of rejection from a partner following disclosure of HIV-positive status:** Majority of the participants in our study stated that they were concerned about being rejected by their partners if they disclosed their HIV status because they would pose too much of a risk or would suffer victimisation or harm, especially if they were financially dependent on them. These justifications prompted them to refrain from disclosing, as shown by the following statements:

‘Eish, it would be difficult still, I believe that if I say, I’m HIV positive, he would actually see it as me being a risk to his life than accepting me the way I am, so we have a baby, I see the future, it’s not easy, an easy future.’ (P1, 22 years, Female, 2nd level, Single)

‘I think maybe because of a bad experience I had with HIV status it makes me be cautious and say no.’ (P2, 32 years, Male, Honour’s degree, Single)

‘I’m, no he knows, if he knows, I don’t know, he’s one of those abusive people so might, you know, harm me at some point, and he supports me so I don’t think it will be good, like financially. Now, if I tell him that I am HIV positive, it is something else he is just gonna stop and he is gonna run and I depend on him …’ (P7, 27 years, Female, 3rd level, Single)

#### Theme 2: Antiretroviral packaging challenges

Participants on ARVs complained about the rattling sound made by the container which drew attention and made them anxious about inadvertent disclosure, and recommended improvements. The theme yielded two subthemes which are discussed below.

**Sub-theme 2.1: The antiretroviral container is perceived as discloser of status:** Several comments underlined participants’ fear of the sound drawing unwanted attention:

‘… if you take the container of ARV or a container of just normal pills and shake them, they do not sound the same. And I think people have noticed that … if maybe the containers made the same sounds, it would be better, because either, whether I am carrying ARV or I am carrying just normal pills, no one will know …’ (P1, 22 years, Female, 2nd level, Single)

‘… but then even walking around with the containers Ahhh!. Is a challenge, the noise that the pills make inside the container …’ (P6, 28 years, Female, 4th level, Engaged)

The sound as well as the recognisable name of the medication on the packaging caused concern:

‘… the packaging … it’s very noisy, so maybe the packaging changes or maybe we are provided with trays that don’t have the name because since time is evolving people know the names of the product written on the packaging.’ (P9, 21 years, Female, 3rd level, Single)

In this way, the packaging itself adversely affected the medication schedule when others were present, as one participant explained:

‘… if people did not know the packaging of the ARVs, I wouldn’t have a problem taking the drug public …’ (P7, 27 years, Female, 3rd level, Single)

**Sub-theme 2.2: Recommendations for improved packaging and handling of antiretroviral medication:** Participants in our survey believed that improving ARV packaging would aid in adherence. Making sure they would not experience any trouble from carrying their medication in public would help them take their medications more regularly. The participants talked about how they felt about the way ARVs were packaged and offered several suggestions for improvement, including replacing the container with one that does not make a sound:

‘… if the containers made the same sounds it would be better, because either, whether I am carrying ARV or I am carrying just normal pills, no one will know, no one will notice … eh.’ (P1, 22 years, Female, 2nd level, Single)

‘… you will find that maybe I am coming from the health center to take my medication, when I am walking you hear kgonche! Kgonche [*sound gesture*]. So everyone knows that, oh, ARVs, for me, they, I think if maybe people should change their containers, maybe use something that will not make that sound or maybe something …’ (P3, 28 years, Female, 3rd level, Married)

Further suggestions were made for the container to be changed to blister packs or have medication in the form of injectables to reduce problems associated with the packaging.

‘… the ones they use for contraceptives, the ones they pack individually in a simple pack, plastic bag, I mean that when you open it it’s like you are opening a sweet, even if you open it in front of people it’s rare that someone …’ (P10, 23 years, Female, Honour’s degree, Engaged)

‘… Aahhh nna [*me*] I thought fear that they can just inject us and we know that we can go once, like family planning. So, I think once is better. People will not miss their doses …’ (P6, 28 years, Female, 4th level, Engaged)

Suggestions were also made to anonymise the medication so that it is not recognisable as ARV:

‘… maybe we are provided with trays that don’t have a name because people know the names of the product that is written on the packaging … yes like … those with Sunday, Monday, Tuesday, Wednesday, then you put them there … yes.’ (P8, 21 years, Female, 4th level, Single)

‘… placing them in a different container so that they don’t look like ARVs.’ (P7, 27 years, Female, 3rd level, Single)

#### Theme 3: Service delivery affects adherence to antiretrovirals

Participants stated that they are indirectly exposed to other people because of the way the services are offered in clinics. They claimed that there was no privacy, which made it challenging for them to consult. This theme yielded two sub-themes: (1) secluded area for HIV-positive patients, and (2) the attitude and competence of healthcare providers.

**Sub-theme 3.1: Secluded areas for HIV-positive clients stigmatise users of the service:** Participants felt that secluded rooms or separate areas for consulting with HIV-positive clients stigmatised users of this service. They appreciated the service they received at the Student Health Centre where positive clients on ARVs were not secluded from others. They approved of this practice as a way of reducing the risk of stigma:

‘… so on campus it is very better because there is privacy, and when you consult nobody asks you if you are going to take ARVs, so you just go straight and consult, that’s it, but then … in the communities HIV-positive people are stigmatized, they always say HIV-positive people this side, mothers of new babies this side, they actually seem to classify us and it’s not good.’ (P1, 22 years, Female, 2nd level, Single)

‘The treatment in the health center is good as everyone welcomes us, they don’t criticize us to say “hey wena [*you*] with HIV go that side,” they just keep it confidential unlike our clinics at home.’ (P6, 28 years, Female, 4th level, Engaged)

One of the students verified that they discriminate against HIV-positive people and dedicate special areas to them at their local clinics and hospitals:

‘… they discriminate people who are HIV positive, then you find that there is a place just for people who are HIV positive, maybe there is a building for all people who are HIV positive … So, the treatment that you could get or have from the Health Centre is different from the one you get from the other …’ (P3, 28 years, Female, 3rd level, Married)

**Sub-theme 3.2: The attitude and competence of healthcare providers:** The attitude and comprehension of the staff at the Student Health Centre during consultations received praise from the study’s participants.

They expressed distress when staff in other health facilities nearer to home seemed discriminatory or judgemental, which made them decide to miss follow-up visits in order to avoid such unpleasantness:

‘… the staff [*at the Student Health Center*] also do not judge you and don’t give you the look that you are HIV-positive, how did you get it, you know, try and become defensive about why you are positive, right they would be giving you looks and those looks are not nice, hence sometimes I don’t go when I’m home, you know … I would wait for you to come back to school.’ (P7, 27 years, Female, 3rd level, Single)

‘Sometimes when I’m home, I feel like eish why can I maybe study forever or until I die, because of the treatment I get here, eish I don’t know how to compare it with other health facilities because you will find that they discriminate people who are HIV positive. Even if they are staff members, they are health professionals, you find that they discriminate and then you find that they don’t, I forgot this word, how to sort of … they don’t respect people who are HIV positive. They think if you are HIV positive, it means you were sleeping around …’ (P3, 28 years, Female, 3rd level, Married)

One of the participants explained the beneficial effect that compassionate staff attitudes can have on patients’ adherence:

‘… I know the importance of taking my medication and also the health professionals around here, they make it easier for us to collect our medication, because like, during our collection of medication you can feel like the person you are consulting like you can relate to the person.’ (P5, 30 years, Male, Master’s degree, Married)

### Phase 2

The second phase follows-up on the participants who were interviewed in the first phase, to find out whether and to what extent the conditions during pandemic restrictions had affected their experiences and perspectives. During COVID-19, students were asked to vacate their residences and had to go to their respective homes abruptly as it was hard lockdown. The university was closed and only in July 2020 did they start with online learning. This was a difficult time for everyone as they had to adjust. Students on treatment were given ARVs for 3 months, not knowing how long the lockdown would last. This meant them having to go to their local clinics, without even having the proper transfer documents. They had to explain themselves and their status and others were not ready to disclose but were forced to. At the local clinics, they had to queue up for hours and even then they were not certain that they will get assistance. Only 3 of the original 10 participants could be reached for interviews; the rest were no longer in the system anymore or their contact details were now out of date. The phase 1 data collection procedures were replicated and participants’ responses were recorded in the same way. The same semi-structured interview guide was used to establish whether or not the participants had experienced the same challenges during the 2 years of COVID-19. Three themes emerged from the data analysis: (1) Non-disclosure to family members and fear of rejection. (2) Antiretroviral packaging and indirect disclosure of HIV-positive status. (3) Undesirable service delivery in local clinics.

#### Theme 1: Non-disclosure to family members and fear of rejection

Participants still felt that they were unprepared to tell their family members about their HIV-positive status, and found it difficult to do so. This made it difficult for them to adhere to the strict time schedules for taking treatment as required. They still feared that their family members would shun them if they found out about their HIV-positive status, especially in the context of the pandemic. Lockdown and the sudden drastic changes in living conditions that it brought, made it difficult for them to disclose. They were unprepared, unsure how the family would react, and fearful of being chased away from home, with nowhere else to go during the restrictions:

‘The lockdown was hard for me and I couldn’t tell my family members because I didn’t know how they would react, what if they chased me away?’ Where was I going to go? Remember that not everyone understands the mode of HIV transmission; it is not a topic for discussion in my family.’ (P1, 22 years, Female, 2nd level, Single)

‘I was not ready to share my status with any of my family members yet, the lockdown happened so quickly and there was no time to prepare. Disclosure, I was told during counseling, is a process and you have to be ready, and I was not. You know the fear of the unknown? I had that because I did not know how my family would react.’ (P2, 32 years, Male, Honour’s degree, Single)

One participant, however, shared the experience of the new pressure she felt to disclose to her family and, being overwhelmed by the love and support she received:

‘I had no choice but to disclose as I did not know how long the lockdown was going to last. Imagine if it was the whole year and I had to go get my medication or go for a blood review or I was sick, how would I cope if I did not tell them? I was ready for any reaction and to my surprise they embraced me and you cannot imagine the love and support I am getting, especially my mother, she even reminds me of the time and I laugh with her, because I have now mastered that. I support disclosure as it makes things easier, now I can take my medication freely and on time, even. She even went to the clinic with me for collection, just to make sure no one mess with me [*laughing*].’ (P3, 28 years, Female, 3rd level, Married)

#### Theme 2: Antiretroviral packaging and indirect disclosure of HIV-positive status

The participants reported that, even during pandemic-induced conditions which reduced their interaction with others in public, they would still decant the medication into other forms of packaging. This led them to miss their time schedules to take the drugs or even skip their doses:

‘I always take my medications out of the container as I cannot stand the rattling or crackling sound it makes. When it is time to take my medication, it will be in a plastic package and no one has to hear anything at home. I sleep in the same room as my two sisters and during lockdown I had to take my medication very discreetly, the plastic container does not make a sound … sometimes they don’t sleep and I end up sleeping without taking my pill because it will be too late to take my dose.’ (P1, 22 years, Female, 2nd level, Single)

‘That container neh, my pills are stuffed with cotton wool and that makes the sound quiet. I wait for everyone to go to sleep before I can take my medications and I must admit, it is not at the same time, but then what can I do? I don’t think that period allowed me to tell them, as we were already stressed by the lockdown and now to bring news like that to the family, which would not have worked for me.’ (P2, 32 years, Male, Honour’s degree, Single)

#### Theme 3: Undesirable service delivery in local clinics

Two of the participants reported deep frustration during the lockdown. The sudden change in living conditions gave them no time to take transfers to the local clinic, and they did not know how long the supply they had would last. The university was in lockdown and brought reliance on local clinics for students’ ARV supplies; but the new conditions brought challenges in accessing medication during the national state of emergency. The participants report that, when they moved to local clinic, they were sent from one nurse to another and even from manager to manager, as they did not have transfer letters, but their green cards meant they were helped, although the reception they received often felt inadequate and they felt exposed and vulnerable. One of the participants explained having to take the risk of travelling without a permit to the university health centre for more caring service:

‘Remember that we thought the lockdown was for a short while, I did not have a transfer letter and when I ran out of pills, the only thing I had to do was pretend that I am sick so that my family would not be suspicious. When I got to our clinic, I had to explain my situation to the nurses so that they could help me, but they will take you up and down before you get help. Luckily, I had my green card so I would just give 1 month of supply and that meant going to the clinic every month. I had to lie to my family that they told me to come for reviews because I had gynecological problems and they wouldn’t ask much after that.’ (P1, 22 years, Female, 2nd level, Single)

‘Our clinics still have those separate areas for HIV patients and it was very difficult for me because it will mean that someone in the community would see me in the queue there. I risked and called one of the sisters at the Health Center here [*at the university*] and I would risk travelling to the university without a permit because I was not going to let my secret out. The Sister assisted me and even made sure I got a permit from the police station.’ (P2, 32 years, Male, Honour’s degree, Single)

The pandemic brought curtailed services, which added uncertainty:

‘… There was no blood monitoring during Covid-19 as all services were interrupted, I just took my medications and hoped for the best.’ (P2, 32 years, Male, Honour’s degree, Single)

One of the participants reported the advantage of having family support when going to the local clinic which, although expensive, helped to remove difficulties associated with adherence to treatment:

‘It was easy for me to take my medications since my mom was with me all the time, no one dared to mess with me. Because I did not have the transfer letter it meant I had to go there every month and the up and down was a bit expensive for both of us.’ (P3, 28 years, Female, 3rd level, Married)

## Discussion of findings

The study’s objective was to investigate HIV-positive individuals whose viral load values were discovered to be unsuppressed in order to identify any potential underlying causes for these surprising outcomes.

This section presents the discussion of those findings under the following themes: disclosure versus stigma and difficulties with treatment when among people; ARV packaging challenges; and service delivery affects adherence to ARVs.

Because of their fear of being judged and excluded as a result of the perceived stigma associated with their HIV status, participants avoided telling their roommates about their status. They acknowledged that it was problematic for students to tell their friends that they had HIV, and that doing so seriously jeopardised their adherence to treatment. According to research conducted in Uganda by Bikaako-Kajura et al. (2006), stigma and not disclosing one’s HIV status to those with whom one lives have a direct bearing on whether or not treatment is successful. The responses from students in the current South African study support that finding.

Marukutira (2012) confirmed that having the support of friends, family, and the community helps teenagers adhere well to their medication schedule. This network of support significantly increased treatment compliance.

The students found it difficult to tell their families that they were HIV-positive, which prevented them from taking their medication in front of them because they were afraid of being disapproved of or judged. In spite of talks and education in the media and healthcare facilities, Ndou, Risenga and Maputle (2013) found that families are frequently unaware of the mode of transmission of HIV and continue to harbour prejudice against HIV-positive family members. The study focused on the experiences of HIV-positive patients receiving ARV in Limpopo. Other students reported being expelled from home after their parents discovered their HIV-positive status.

Participants in the study feared of being accused of infidelity and unfaithfulness. However, studies in developed and developing countries have shown that disclosures to sexual partners is increasing. However, majority of those in these studies who disclosed their identities reported kindness, improved social support, acceptance, and strengthened relationships. Some people still reported fear of negative effects such as rejection, blaming, abandonment, and stigma. However, Ndou et al. (2013) found that their Limpopo participants had difficulty disclosing to their partners because of the risk of being blamed, loss of economic support, abandonment, physical and emotional abuse from partners, discrimination, and disruption of family relationships. The students in the current study did not report the encouraging range of experiences. The participants also worried about being accused of unfaithfulness and infidelity. In the USA, Parsons et al. (2004) also detailed unpleasant incidents related to disclosing one’s positive HIV status to a partner.

The participants’ comments in phase 2 revealed the pressure that the state of emergency placed on them to consider disclosure, given their dependence on accommodation at home when normal university living conditions were suddenly disrupted. When these conditions arose suddenly to make it impossible to avoid disclosure, it revealed unconditional love and support from family that had previously seemed so unlikely; this finding illustrates the unexpected reward for overcoming fear of disclosing to family members.

The participants were concerned that the noise made by the medication in its container would give away their HIV-positive status to those around them. They believed that others can recognise the distinctive sound made by a container with HIV medication, and they perceived that this noise would indirectly disclose their status.

In a study conducted in Botswana, Geiselhart et al. (2015) expressed concern about the lack of literature on the impact of ARV packaging on persons living with HIV and/or AIDS. Schlatter, in particular, has seen persons living with HIV and/or AIDS getting irritated by the rattling noise the tablets make inside the plastic bottle in the course of his studies on ARV adherence. This was said to be worse while utilising public transportation because other passengers would know it was ARVs because the sound stigmatises them.

In Nigeria, Sekoni, Obidike and Balogun (2012) concurred with the aforementioned remark, noting that patients’ selected coping technique for averting stigma was to take ARV from the labelled containers and place them in unmarked pill boxes.

Service delivery points for HIV that are isolated from others were considered among our participants as a way of discriminating and indirectly disclosing patients’ status.

The impact that this might have on treatment was confirmed by a study conducted in India, which identified service factors and the standard of care as potential barriers to adherence, with discrimination among clients at the point of service delivery as a significant hurdle when treating HIV and/or AIDS (Beattie et al. 2012).

The purpose of the current study was to emphasise the value of compassionate and understanding clinic staff for HIV-positive patients. Murphy et al. (2000) found that patient satisfaction with healthcare, the relationship between adult clients and healthcare providers, and the effectiveness of communication between clients and healthcare providers, all had a substantial impact on adherence. Participants who had favourable relationships and good communication with healthcare professionals reported taking their medication as directed, whereas those who had negative encounters with healthcare professionals reported low levels of adherence.

A study by Molelekwa (2020), who wrote about the difficulties in getting access to ARVs during lockdown and found that getting treatment and ARVs was difficult during the national lockdown, echoed the frustrations of the students who were unable to access the university clinic during that time. There were commonalities between the two stages of the study in that the clients were coerced into disclosing their status without being given the option to do so.

Students who had to collect medication from the community clinics still suffered the same fate and highlighted that the container for ARVs is actually the one that draws attention, as community members are aware of the sound made, and this is causing stigma.

According to a study done in KwaZulu-Natal, personnel need to be trained on adherence counselling and in-service education on compliance strenghtened to ensure effective ART adherence. To make sure that adherence was successful, adequate staffing was also required. This is done to make sure that patients using ART are monitored and that virological failure, which can result in drug resistance, is both identified and treated (Uzidike et al. 2015). Other observations showed that the staff-to-patient ratio made it difficult to keep track of the patients’ virological monitoring. According to the researchers, reducing the staff deficit will enhance counselling and doctor–patient communication because patients will have more time to talk to caregivers and can address their worries and fears (Chabikuli et al. 2010).

## Recommendations

The findings from phases 1 and 2 emphasise the need of enhancing the social context of disclosure and imply the necessity for initiatives designed to lessen stigma in order to enhance a safer environment for disclosure:

- Among the recommendations emerging from the study was that pharmacists could improve ARV packaging, as its recognition as containing treatment for HIV-positive patients was seen as indirectly revealing their status and stigmatising them, causing barriers to adherence.
- Students in our study also advised using a supermarket model of service delivery as a method to receive all the aid they require without disclosing their status, so lowering the danger of stigma.
- The results also revealed that in order to address the issue of stigma and improve adherence, the general public and communities needed to be better informed and sensitised on issues surrounding HIV and/or AIDS. Campaigns to raise awareness and the distribution of informational pamphlets to the entire neighbourhood could accomplish this. Aside from talks with all interested parties, outreach initiatives can be implemented in places where communities congregate. In colleges, student representative councils can act as the voice of and for the students. Healthcare professionals should have the necessary training and resources to give patients all the assistance they require in a way that hides their status. Participants also proposed user-friendly packaging.

## Conclusion

The experiences of students whose viral load did not decrease despite taking ARVs for more than a year were examined in this study. The results from both phases point to the students’ perception that they could not reveal their status to their family members, roommates, or partners as one of the major causes of virological failure. As a result, it was challenging for them to take their medication when among other people. This reluctance results in noncompliance with the rigid schedule demanded by the medication regimen.

Students clearly identified the problems they have when it comes to what affects their adherence to medication, challenges, and what can bring about success in that effect. This is a population that is not researched greatly and only the adult population has been looked into. This study highlights the challenges experienced by young people when it comes to achieving virological success.

### Limitations and future research

This qualitative exploratory study has identified clear challenges experienced by students on ARV treatment whose viral load remained high. Because the sample was small and was limited to students from the university of Limpopo, the findings are not transferable, and are not necessarily representative of a wider South African student population. There is scope to examine the relevance of these findings to other student groups in the country, to compare them, and to use material from larger studies to guide targeted interventions that could improve adherence to treatment by young people. The findings also offer a starting point for further research into, for example, the experiences of roommates or family members living with an HIV-positive person on HAART after having disclosed their status, the views of pharmaceutical companies on the packaging of ARVs for greater discretion, and the views of service providers about improving problematic features of healthcare care provision for HIV-positive patients.
